# Effects of diabetes mellitus during pregnancy on pregnancy outcomes and growth and development of children aged 0–6 years: a retrospective cohort study

**DOI:** 10.3389/fendo.2026.1811874

**Published:** 2026-08-21

**Authors:** Tianzi Li, Lili Ma, Jun Gang, Feng Liang, Xiuhua Ma, Juan Zhang, Meng Wang

**Affiliations:** 1 Department of General Practice, The Second Affiliated Hospital of Zhengzhou University, Zhengzhou, China; 2 Department of Obstetrics and Gynecology, Capital Medical University Daxing Teaching Hospital, Beijing, China; 3 Department of Obstetrics and Gynecology, Beijing Daxing District Maternal and Child Health Hospital, Beijing, China; 4 School of Statistics, Beijing Institute of Petrochemical Technology, Beijing, China

**Keywords:** diabetes mellitus during pregnancy, gestational diabetes mellitus, pregnancy outcome, offspring, growth, children

## Abstract

**Background:**

Gestational diabetes mellitus (GDM) and pre-gestational diabetes mellitus (PGDM) are jointly termed as diabetes mellitus during pregnancy. This condition exhibits a relatively high incidence rate and exerts adverse impacts on both the mother and the fetus.

**Objective:**

To investigate the differential pregnancy outcomes and offspring development between pregnant women with and without diabetes mellitus during pregnancy.

**Methods:**

A retrospective cohort study was carried out in China. From 2014 to 2016, all pregnant women in a specific region of Beijing who met the exclusion criteria, along with their offspring who had undergone physical examinations at ages ranging from 0 to 6, were selected as the research subjects between 2014 and 2023. We analyzed the outcomes separately for women with PGDM and GDM. SPSS 27.0 and R 4.2.2 were used to analyze the data.

**Results:**

This three-year retrospective study included 49,912 deliveries from a specific region of Beijing, among which 49,765 were live births. The overall prevalence of diabetes during pregnancy was 8.6%, comprising 4,003 cases of GDM and 284 cases of PGDM. PGDM significantly increased the risks of neonatal hypoglycemia (*OR* 17.344, 95% *CI* 11.703–25.703) and neonatal infection (*OR* 8.043, 95% *CI* 5.584–11.585) (*p* < 0.001). GDM was identified as an independent risk factor for multiple adverse pregnancy outcomes. Notably, the strongest associations were observed for neonatal hypoglycemia (*OR* 7.299, 95% *CI* 5.844–9.116), pathological jaundice (*OR* 7.264, 95% *CI* 5.923–8.910), neonatal infection (*OR* 2.876, 95% *CI* 2.415–3.424), asphyxia neonatorum (OR 2.435, 95% CI 1.802–3.291), macrosomia (*OR* 1.355, 95% *CI* 1.217–1.508), and cesarean section (*OR* 1.207, 95% *CI* 1.131–1.288) (all *p* < 0.001). This study statistically analyzed 18, 590 pairs of mothers and children who had completed the national standard health check-up for children aged 0 to 6. Newborns in the diabetes during pregnancy group exhibited significantly higher body length and weight at birth compared to those in the non-diabetes group (*p* < 0.001), However, from 3 to 72 months of age, the body length and weight distributions of children in both groups gradually converged, with the differences diminishing over time.

**Conclusions:**

Diabetes mellitus during pregnancy is associated with a substantially increased risk of adverse outcomes for both the mother and the neonate. Nevertheless, the influence on childhood physical development remains indeterminate. Strengthened screening and management of high-risk pregnant women, particularly those with diabetes, are of critical importance for reducing the incidence of adverse pregnancy outcomes and enhancing the health of both mothers and their progeny.

## 1 Introduction

Diabetes mellitus during pregnancy encompasses both pre-existing diabetes and gestational diabetes mellitus (GDM), with the latter accounting for over 85% of cases (1). When diabetes complicating pregnancy coexists with pregnancy, it is termed “pre-existing gestational diabetes mellitus (PGDM)”; whereas when normal glucose metabolism occurs before pregnancy and develops during gestation, it is classified as GDM (2). Notably, GDM often persists beyond delivery. Throughout pregnancy, mothers undergo a series of metabolic changes. GDM is a high-risk pregnancy condition. It exposes fetuses to prolonged intrauterine hyperglycemia, which may adversely affect maternal organ function, maternal-fetal outcomes, and even children’s health during childhood (3, 4). Recent studies have confirmed that GDM is associated with a range of adverse pregnancy outcomes, including miscarriage, stillbirth, preterm birth, congenital anomalies, postpartum hemorrhage, and premature rupture of membranes, macrosomia, and neonatal hypoglycemia (5), significantly increasing risks of maternal and fetal complications. The adverse effects of intrauterine hyperglycemia on offspring may persist into adulthood (6). Consequently, considering the substantial effects of GDM on pregnancy outcomes, including increased incidences of macrosomia, fetal growth restriction, miscarriage, preterm birth, and congenital anomalies, along with long-term health hazards for the offspring, it is of utmost importance to investigate these effects and formulate appropriate interventions for clinical practice.

## 2 Data and methods

### 2.1 Participants

All pregnant women and their offspring who received routine antenatal examinations and delivered successfully in a district of Beijing from 2014 to 2016 were included in the study cohort.

The inclusion and exclusion criteria are presented as follows:

Inclusion criteria for pregnant women: those who have registered for antenatal care and undergone regular prenatal examinations; those who have given birth within the region. Exclusion criteria: those who have had fewer than five prenatal examinations; those who have only had prenatal examinations but did not give birth in this region.

Inclusion criteria for children: born within this district; mothers meet the maternal inclusion criteria; have completed the national standardized physical examinations for children aged 0–6 years during the period from 2014 to 2023, characterized by a high rate of health screenings and management. Exclusion criteria: those who have not completed the national standardized physical examinations for children aged 0–6 years.

### 2.2 Study design

**Figure 1:**
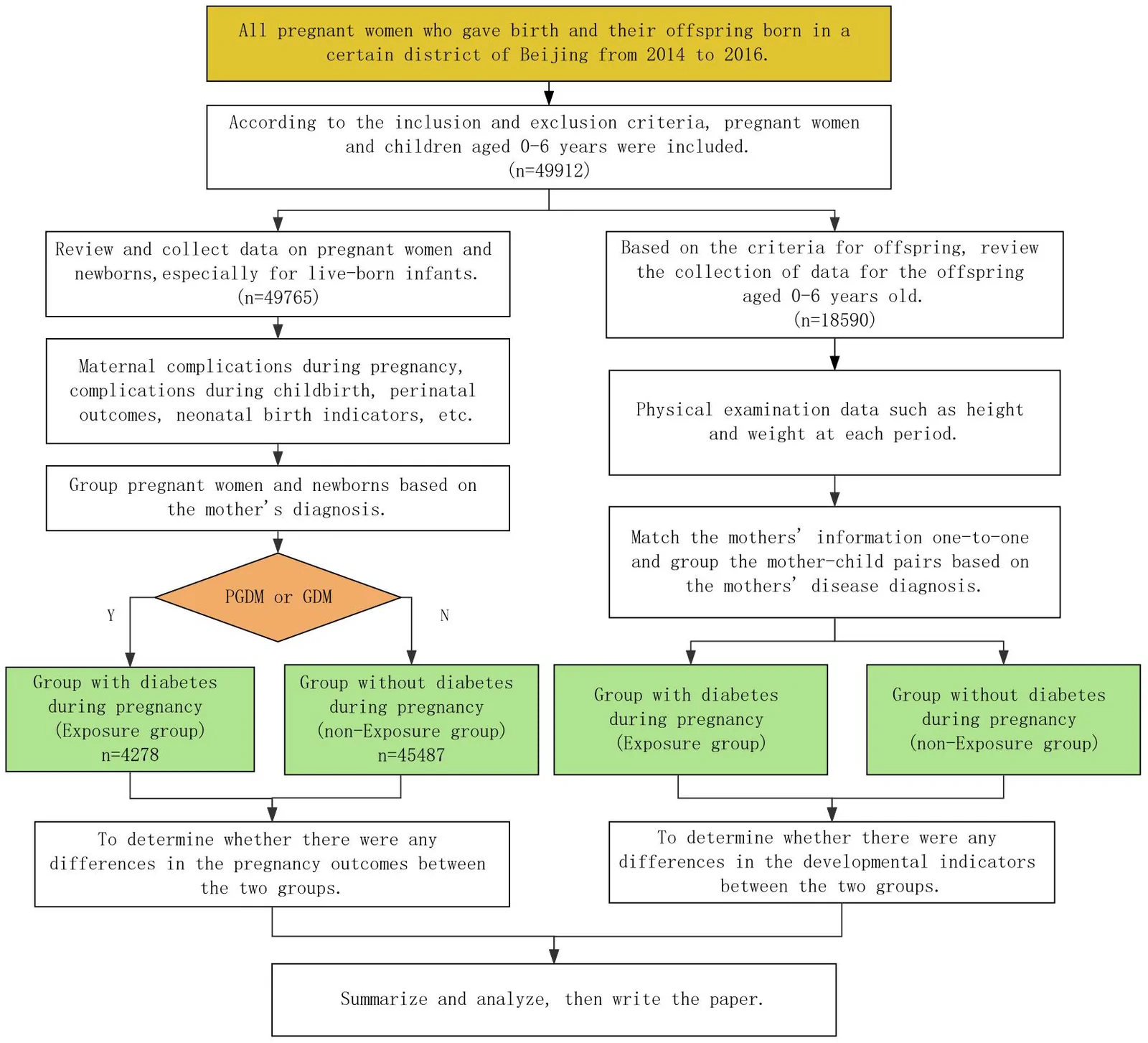
Overview of the study design and analytical workflow.

The overall study design and analytical workflow are depicted in **Figure 1**.

Collect basic information from all pregnant women. Gather pregnancy-related data including maternal age, pregnancy stage, number of previous pregnancies, and presence of chronic comorbidities. Determine diagnoses of GDM or PGDM. To diagnose the disease, healthcare providers should follow the established diagnostic criteria, which include conducting an oral glucose tolerance test (OGTT) between 24 to 28 weeks of pregnancy for GDM (7), and considering pre-pregnancy diabetes status and specific blood glucose levels for PGDM. Using pregnant women or mothers with diabetes as the exposure factor, pregnant women with PGDM and GDM were classified into the group of diabetes mellitus during pregnancy (exposure group), whereas those without diabetes are categorized as the non - exposure group.

Participants with incomplete pregnancy outcome records, missing key data, or loss to follow-up were excluded during data cleaning. Collect data encompassing stillbirth rates, preterm birth rates, full - term delivery rates, and cesarean section rates. Gather data on pregnancy outcomes, with a focus on perinatal outcomes, delivery methods and complications, as well as neonatal outcomes, including complications affected by placental abruption. Outcome definitions were standardized in accordance with World Health Organization (WHO) criteria. Preterm birth was defined as delivery prior to the completion of 37 weeks of gestation. Standardize the collection of children’s birth records and community health check - up data for children aged 0 to 6 years. This collection encompasses the documentation of gender, height measurements at various developmental stages, and weight indicators. Growth status was assessed using age- and sex-specific z-scores based on the WHO Child Growth Standards (8) and the Chinese national growth standards for children under 7 years of age (WS/T 423-2022) (9).

### 2.3 Data sources and ethical principles

The data utilized in this study were sourced from the medical record systems of Beijing’s maternal and child healthcare system, as well as eligible obstetric hospitals or maternal and child health centers within the region. The research has obtained ethical approval from the Ethics Committee of Daxing District People’s Hospital in Beijing (Approval Number: No.20240513KY-1-02) and has been exempted from the requirement of informed consent. In strict compliance with confidentiality principles, the study implemented data anonymization measures. Hereby, we pledge to utilize all collected information exclusively for population analysis, ensuring the highest level of confidentiality for the participants. Unauthorized access to any related information by unauthorized personnel is strictly prohibited.

### 2.4 Statistical methods

Data collection was performed using Epidata 3.1 and Excel 2010, with data entry conducted independently by two researchers to minimize errors. Logical validation was performed prior to statistical analysis using SPSS 27.0 and R 4.2.2.

Descriptive statistics were presented as frequencies and percentages for categorical data. Normally distributed continuous variables were expressed as mean ± standard deviation (SD), while skewed data were summarized as median with interquartile range [M (IQR)]. Between-group differences in continuous variables were assessed using Student’s t-test or the Mann-Whitney U test, whereas categorical variables were analyzed using the chi-square test or Fisher’s exact test, as appropriate. Maternal and offspring data were matched using R software.

To investigate the impact of diabetes during pregnancy on maternal outcomes and adverse neonatal events, we applied a combination of descriptive statistics, chi-square tests, binary logistic regression, and quantile regression models. For longitudinal growth and developmental outcomes in offspring, we utilized descriptive statistics, the Wilcoxon rank-sum test, chi-square tests, and linear mixed-effects (LME) models. All statistical tests were two-sided, and a p-value < 0.05 was considered indicative of statistical significance.

## 3 Results

### 3.1 Basic data analysis

From 2014 to 2016, a district in Beijing recorded 49,912 eligible deliveries. The maternal age ranged from 27.85 ± 4.362 years, with pregnancy duration (1.88 ± 1.113) and childbirth frequency (1.35 ± 0.524) measured in weeks. The gestational age at delivery averaged 39.28 ± 1.573 weeks, with 49,555 cases being single pregnancies.

There were 4,287 cases of diabetes mellitus during pregnancy, with an incidence rate of 8.6% (4,287/49,912). Within this diabetic group, GDM was the predominant subtype, accounting for 93.4% (4,003/4,287) of cases. The remaining 6.6% (284/4,287) were PGDM cases, comprising 279 with type 2 diabetes and 5 with type 1 diabetes. Of the 49,765 live births, 4,278 were diabetes mellitus during pregnancy, with an incidence rate of 8.6%. Non-diabetic mellitus was 45,487 cases, accounting for 91.4%.

In the overall cohort of 49,482 pregnant women, GDM accounted for 8.09% (4,003/49,482) of the study population. The remaining women were classified as the non-GDM unexposed group.

The adverse outcomes of perinatal pregnancy encompassed stillbirth, with a significant incidence rate. The live birth rate was 99.7%, the preterm birth rate was 0.9%, and the full-term birth rate was 98.5%. The vaginal delivery rate was 56.8%, and the cesarean section rate was 43.2%.

A total of 49,765 live births were included. The prevalence of macrosomia was 8.1% (4,032 cases), while low birth weight and extremely low birth weight accounted for 3.1% (1,567 cases) and 0.4% (184 cases), respectively.

A total of 18,590 children aged 0–6 years (representing 18,590 mother-child pairs) who met the inclusion criteria were included in the final analysis.

### 3.2 Effects of diabetes on perinatal outcomes

In the analysis of 49,912 pregnant women, 16 cases in the exposed group developed adverse perinatal outcomes. A chi-square test conducted on intrauterine hyperglycemia and its association with adverse perinatal outcomes revealed a slightly elevated incidence rate in the group exposed to intrauterine hyperglycemia compared to the unexposed group (0.4% vs 0.3%, χ²=1.461, *p* > 0.05), yet this difference did not reach statistical significance.

### 3.3 Impact of PGDM on pregnancy outcomes

Utilizing diabetes during pregnancy as the independent variable(binary variable, 0= no, 1= yes), the study employed logistic regression to assess its impacts on pregnancy outcomes. Due to the limited number of women with type 2 diabetes mellitus (n = 5), stratified analyses comparing type 1 and type 2 diabetes against the non-diabetic reference group were not performed to avoid insufficient statistical power and model overfitting.

Each independent variable was entered separately into a binary logistic regression model to identify its association with PGDM. As shown in **Table 1**, the odds ratios (OR) for C-sect (*OR* 0.769, 95% *CI* 0.604-0.980), postpartum hemorrhage (*OR* 0.427, 95% *CI* 0.305-0.598), and placenta previa occurrence (*OR* 0.387, 95% *CI* 1.112-1.950) were observed statistically significant (*p* < 0.05).

**Table 1:** Multivariable logistic regression analysis of the association of PGDM with adverse maternal and infant outcomes (n=49765, Model 1*).

Outcome	*B*	*S.E.*	Waldχ²	*p*-value	*OR* value (95% *CI*)
Natural labor	0.215	0.122	3.115	0.078	1.240(0.976-1.576)
C-sect	-0.260	0.123	4.450	0.035	0.771(0.605-0.982)
Premature birth	0.128	0.582	0.049	0.826	1.137(0.363-3.559)
Postpartum hemorrhage	0.853	0.172	24.700	<0.001	2.346(1.676-3.284)
Premature rupture of fetal membranes	0.030	0.244	0.015	0.902	1.030(0.638-1.663)
Placenta praevia	0.951	0.385	6.091	0.014	2.589(1.216-5.512)
Placental abruption	1.528	0.299	26.175	<0.001	4.608(2.566-8.273)
Fetal distress	0.057	0.238	0.057	0.811	1.059(0.664-1.689)
Macrosomia	-0.045	0.223	0.042	0.839	0.956(0.618-1.479)
Birth defects/congenital malformations	0.681	0.584	1.359	0.244	1.975(0.629-6.200)
Infection of newborn	2.085	0.186	125.409	<0.001	8.043(5.584-11.585)
Asphyxia neonatorum	0.573	0.583	0.966	0.326	1.774(0.566-5.567)
Pathological jaundice of newborn	1.690	0.299	31.900	<0.001	5.420(3.015-9.743)
Hypoglycemia of newborn	2.853	0.201	202099	<0.001	17.344(11.703-25.703)

*Adjusted for age, gravidity, parity. Each outcome modeled separately with PGDM as exposure.

Multivariable logistic regression analysis (**Table 1**) demonstrated that PGDM was significantly associated with an increased risk of postpartum hemorrhage (OR 2.346, 95% *CI* 1.676–3.284), placenta praevia (*OR* 2.589, 95% *CI* 1.216–5.512), placental abruption (*OR* 4.608, 95% *CI* 2.566–8.273), neonatal infection (OR 8.043, 95% *CI* 5.584–11.585), pathological jaundice (*OR* 5.420, 95% *CI* 3.015–9.743), and neonatal hypoglycemia (*OR* 17.344, 95% *CI* 11.703–25.703) (all *p* < 0.05). Conversely, PGDM showed a significant negative association with cesarean section (*OR* 0.771, 95% *CI* 0.605–0.982, *p* = 0.035). No statistically significant associations were found for the other maternal and neonatal outcomes.

### 3.4 Impact of GDM on pregnancy outcomes

After excluding PGDM cases, we analyzed the impact of whether the pregnant women had GDM on the pregnancy outcome. The specific regression analysis results are presented in **Table 2**.

**Table 2:** Multivariable logistic regression analysis of the association of GDM with adverse maternal and infant outcomes (n=49482, Model 2*).

Outcome	*B*	*S.E.*	Waldχ²	*p*-value	*OR* value (95% *CI*)
Natural labor	-0.193	0.033	33.998	<0.001	0.825(0.773-0.880)
C-sect	0.188	0.033	32.395	<0.001	1.207(1.131-1.288)
Premature birth	-0.432	0.206	4.386	0.036	0.649(0.433-0.973)
Postpartum hemorrhage	-0.331	0.076	19.141	<0.001	0.718(0.619-0.833)
Premature rupture of fetal membranes	-0.073	0.070	1.063	0.303	0.930(0.810-1.068)
Placenta praevia	0.261	0.152	2.959	0.085	1.298(0.964-1.747)
Placental abruption	-0.799	0.241	10.990	<0.001	0.450(0.281-0.721)
Uterine rupture	-0.845	0.721	1.377	0.241	0.429(0.105-1.763)
Fetal distress	-0.101	0.070	2.075	0.150	0.904(0.788-1.037)
Low birth weight infant	-0.091	0.098	0.868	0.351	0.913(0.753-1.106)
Very low birth weight infant	-0.693	0.418	2.673	0.102	0.505(0.223-1.145)
Macrosomia	0.304	0.055	30.895	<0.001	1.355(1.217-1.508)
Birth defects/congenital malformations	-0.137	0.239	0.331	0.565	0.872(0.546-1.391)
Infection of newborn	1.056	0.089	140.772	<0.001	2.876(2.415-3.424)
Asphyxia neonatorum	0.890	0.154	33.586	<0.001	2.435(1.802-3.291)
Pathological jaundice of newborn	1.983	0.104	362.394	<0.001	7.264(5.923-8.910)
Hypoglycemia of newborn	1.988	0.113	307.154	<0.001	7.299(5.844-9.116)

*Adjusted for age, gravidity, parity, and pre-pregnancy comorbidities. Each outcome modeled separately with PGDM as exposure.

Multivariable logistic regression analysis revealed that GDM was significantly associated with an increased risk of cesarean section (*OR* 1.207, 95% *CI* 1.131–1.288), macrosomia (*OR* 1.355, 95% *CI* 1.217–1.508), neonatal infection (*OR* 2.876, 95% *CI* 2.415–3.424), asphyxia neonatorum (*OR* 2.435, 95% *CI* 1.802–3.291), pathological jaundice (*OR* 7.264, 95% *CI* 5.923–8.910), and neonatal hypoglycemia (*OR* 7.299, 95% *CI* 5.844–9.116), all with *p* < 0.001.

### 3.5 Effects of diabetes during pregnancy on fetal developmental indicators

We evaluated all mothers with gestational diabetes during pregnancy and their offspring. The Wilcoxon rank sum test (with continuity correction) was employed to compare differences in body length/height and weight distribution between offspring of pregnant women with diabetes and those without diabetes across different age stages. The study indicate that no difference in early anthropometric growth trajectories. At birth, newborns of diabetic mothers exhibited significantly greater body length and weight compared with those of non-diabetic mothers (all *p* < 0.001). However, from 3 to 72 months of age, no statistically significant differences were observed between the two groups in height or weight distribution (all *p* > 0.05).

### 3.6 Model analysis based on R language

#### 3.6.1 Impact of diabetes on pregnancy outcomes

##### 3.6.1.1 Research design and model setting

Variable Definitions and Model Specification.

Dependent variables: Two sets of outcomes were assessed. First, overall adverse pregnancy outcomes in offspring, defined as the presence of any adverse infant outcome included in this study. Second, macrosomia (large for gestational age), which was analyzed independently because it accounted for the highest proportion of all adverse outcomes and exerted a pronounced influence on the results.

Independent variables of interest: The primary exposure variable was diabetes during pregnancy, coded as a binary variable (0 = no, 1 = yes). Additionally, a maternal risk score (HighRisk) was calculated, defined as the cumulative number of risk factors present in the pregnant woman (including GDM, chronic hypertension, and anemia during pregnancy etc.).

Control variables: The following covariates were included to adjust for potential confounding: maternal age, gestational age at delivery, parity, and infant sex.

Statistical model: Given the binary nature of the outcome variables, a binary logistic regression (logit) model was used for the statistical analysis. The model is specified as follows:


$$\text{logit}(P(Y=1|\text{X}))=\ln\left(\frac{P(Y=1|\text{X})}{1-P(Y=1|\text{X})}={\text{β}}^{T}\text{X}\right])$$

where P(Y = 1∣X)P(Y = 1∣X) represents the probability of the outcome given the set of covariates XX, and ββ denotes the vector of regression coefficients.

##### 3.6.1.2 Model empirical results

Leveraging R language for model analysis to investigate the impact of diabetes. The results of the benchmark model are shown in **Table 3**.

**Table 3:** Effects of diabetes on overall adverse birth outcomes in offspring.

Variable	Adverse birth outcomes (model I)		Adverse outcome (Model II)	
	*β*	*t-statistic*	*β*	*t-statistic*
Nodal increment	0.1652 ***	-134.1630	0.0974 ***	-26.1072
Diabetes Complications	1.6910 ***	13.3562	1.6233 ***	12.1790
Age			1.0258 ***	7.4971
Gravidity			1.0228	1.4866
Parity			0.8433 ***	-5.0905
Observed value	49912		49912	
R^2^ Tjur	0.004		0.005	

*p < 0.05; **p < 0.01; ***p < 0.001.

The results demonstrated that diabetes during pregnancy was significantly associated with adverse pregnancy and neonatal outcomes. Specifically, mothers with PGDM or GDM had a 62.3% higher risk of experiencing these complications compared with their non-diabetic counterparts. Additionally, advanced maternal age was identified as a significant risk factor; each 1-year increment in maternal age was associated with an approximately 2.6% increase in the odds of adverse outcomes.

#### 3.6.2 Effects of diabetes during pregnancy on children

##### 3.6.2.1 The mixed effects model is set as follows:


$$\begin{array}{ll} {\text{y}}_{\text{ij}} & ={\text{β}}_{0}+{\text{β}}_{1}\;{\text{Diabetes}}_{\text{i}}+{\text{β}}_{2}\;{\text{Age}}_{\text{ij}}+{\text{β}}_{3}\;({\text{Diabetes}}_{\text{i}}\times {\text{Age}}_{\text{ij}})\\ +{\text{β}}_{4}\;{\text{Age}}_{\text{ij}}^{2}+{\text{β}}_{5}\;({\text{Diabetes}}_{\text{i}}\times {\text{Age}}_{\text{ij}}^{2})+{\text{β}}_{6}\;{\text{GestationalAge}}_{\text{i}}\\ +{\text{β}}_{7}\;{\text{Parity}}_{\text{i}}+{\text{β}}_{8}\;{\text{Gender}}_{\text{i}}+{\text{β}}_{9}\;{\text{BirthWeight}}_{\text{i}}({\text{or BirthLength}}_{\text{i}})\\ +{\text{u}}_{0\text{i}}+{\text{u}}_{1\text{i}}\;{\text{Age}}_{\text{ij}}+\epsilon_{\text{ij}}. \end{array}$$

Random effect assumption: For each model, assume that the random effects of individuals satisfy:


$$(\begin{array}{c} {\text{u}}_{0\text{i}}\\ {\text{u}}_{1\text{i}} \end{array})∼\text{N}\;((\begin{array}{c} 0\\ 0 \end{array}),(\begin{array}{cc} {\text{τ}}_{00} & {\text{ρ}}_{01}\sqrt{{\text{τ}}_{00}{\text{τ}}_{11}}\\ {\text{ρ}}_{01}\sqrt{{\text{τ}}_{00}{\text{τ}}_{11}} & {\text{τ}}_{11} \end{array})),$$

Among which, ${\text{τ}}_{00}{\text{τ}}_{11}{\text{ρ}}_{01}\epsilon_{\text{ij}}∼\text{N}(0,{\text{σ}}^{2})$denotes the variance of the random intercept, denotes the variance of the random slope, and denotes the correlation coefficient between the random intercept and the random slope. Meanwhile, the residual term of the model satisfies, and is independent of the random effects. The variables are described as follows:

${\text{y}}_{\text{ij}}{\text{ijy}}_{\text{ij}}={\text{Height}}_{\text{ij}}{\text{y}}_{\text{ij}}={\text{Weight}}_{\text{ij}}$: The dependent variable of the child at the time of measurement can represent height (cm) or weight (kg).

${\text{Diabetes}}_{\text{i}}\text{i}$: Indicates whether the mother complicate by diabetes.

${\text{Gender}}_{\text{i}}$: Gender of children (0= male, 1= female).

${\text{Parity}}_{\text{i}}\text{i}$: The number of times the child has been born.

${\text{Gestational Age}}_{\text{i}}\text{i}$: The gestational week (weeks) at which the child was born.

${\text{Birth Length}}_{\text{i}}$: Newborn length (cm), only used for length model.

${\text{Birth Weight}}_{\text{i}}$: Newborn weight (kg), only used for the weight model.

${\text{β}}_{0},\;{\text{β}}_{1},\ldots$: Fixed effect coefficient, reflecting the average influence of each covariate on the dependent variable.

${\text{u}}_{0\text{i}}{\text{u}}_{1\text{i}}\text{i}$ And: respectively represent the random intercept for the child and the random slope for month of age, capturing differences in baseline level and growth rate.

$\epsilon_{\text{ij}}{\text{σ}}^{2}$ The measurement error term or individual error is normally distributed with a variance of ${\text{σ}}^{2}$. In the aforementioned model, the fixed effect component is utilized to assess the impact of gestational diabetes, monthly progression (and its quadratic term), their combined effect, gender, parity, gestational age, and newborn size (length or weight) on the growth and development of offspring. while the random effect part reflects the heterogeneity of different individuals in baseline and growth trend.

##### 3.6.2.2 Analysis of the effects of diabetes on offspring body length/height

In the study employing a mixed effects model, we empirically analyzed the developmental trajectories of offspring’s length/height in two age groups (3–78 months and 3–36 months) to assess the effects of diabetes on infant growth.

The model included covariates such as maternal age, gestational age, newborn length, maternal parity, and neonatal infections, while accounting for interactions between GDM and birth month, as well as the square of birth month. The results, detailed in **Table 4**.

**Table 4:** Effects of diabetes on offspring’s body length/height.

Variable	Height (Model I)		Height (Model II)	
	*β*	*t-statistic*	*β*	*t-statistic*
Nodal increment	42.5535 ***	71.4650	39.3511 ***	67.1924
Diabetes complications	-0.1072	-1.6070	-0.1643 *	-2.1238
Gender x [Female]	-1.3033 ***	-42.2410	-1.3163 ***	-43.5040
Parity	-0.2282 ***	-6.1592	-0.1531 ***	-4.2136
Pregnancy weeks of delivery	-0.0015	-0.1352	-0.0008	-0.0754
Delivery [C-Section]	0.0215	0.6842	-0.0061	-0.1996
Mode of delivery [Other]	-0.0949	-0.6548	-0.1609	-1.1323
Pregnancy age	0.0230 ***	5.2853	0.0243 ***	5.7066
Infection of newborn	0.0766	0.6684	0.1276	1.1356
Newborns length	0.3556 ***	37.4548	0.3675 ***	39.4204
Diabetes× month of age	0.0021	0.5293	0.0097	1.1425
Diabetes× month of age^2	-0.0001	-1.3386	-0.0003	-1.2906
Observed value	211726		139605	
Edge R^2^/Condition R^2^	0.955/0.981		0.935/0.971	

*p < 0.05; **p < 0.01; ***p < 0.001.

The results indicate that within the 3–78 month age range (Model I), maternal diabetes showed no statistically significant negative correlation with offspring’s body length/height (estimate = -0.1072, *p* > 0.05). However, within the 3–36 month range (Model II), this effect reached statistical significance (estimate = -0.1643, *p* < 0.05). To better disentangle the sources of explained variance, we report both the marginal R² (R²_m) and the conditional R² (R²_c). The R²_m represents the variance explained solely by the fixed effects of interest (i.e., the treatment or predictor variables). In contrast, the R²_c represents the total variance explained by both the fixed effects and the random effects structure. The substantial gap observed between the R²_c and R²_m indicates that a large proportion of the data variance is attributable to the blocking/grouping structure, thereby statistically justifying the use of a mixed-effects model over a standard linear regression to avoid pseudoreplication. It indicate that the fixed effects and random effects included in the model significantly contribute to explaining the variance.

##### 3.6.2.3 Analysis of the effects of diabetes on offspring weight

In this study, longitudinal data from two age intervals—3 to 78 months (Model I) and 3 to 36 months (Model II)—were used to investigate the impact of maternal diabetes on offspring weight trajectories beyond 3 months of age. For both models, offspring weight measured after 3 months was designated as the dependent variable. The following covariates were included to adjust for potential confounders influencing growth: gestational age at delivery (and its quadratic term), gestational week, parity, maternal age, neonatal infections, infant sex, and birth weight. The results of these analyses are presented in **Table 5**.

**Table 5:** Effects of diabetes on offspring weight.

Variable	Weight (Model I)		Weight (Model II)	
	*β*	*t-statistic*	*β*	*t-statistic*
Nodal increment	4.9059 ***	26.6748	4.7625 ***	25.7932
Diabetes complications	-0.0241	-0.7662	-0.0303	-0.9562
Pregnancy weeks of delivery	-0.0123 **	-2.7013	-0.0114 *	-2.4949
Delivery mode [C-section]	-0.0003	-0.0265	0.0039	0.3110
Mode of delivery [Other]	-0.0502	-0.8725	-0.0593	-1.0311
Pregnancy age	0.0059 ***	3.3932	0.0055 **	3.1737
Infection of newborn	0.0691	1.5179	0.0778	1.7113
Parity	-0.0398 **	-2.7024	-0.0414 **	-2.8143
Gender of newborns [F]	-0.4660 ***	-38.0093	-0.4789 ***	-39.0765
Neonatal weight	0.5269 ***	38.7945	0.5300 ***	39.0219
Diabetes× month of age	-0.0011	-0.4909	-0.0003	-0.1068
Diabetes× month of age^2	0.0000 *	2.2218	0.0000	1.1793
Observed value	211746		177914	
Edge R^2^/Condition R^2^	0.827 / 0.959		0.841 / 0.957	

*p < 0.05; **p < 0.01; ***p < 0.001; The mode of delivery was natural delivery as the reference group.

Utilizing mixed effects modeling on data from 3- to 78-month-old (Model I) and 3- to 36-month-old (Model II) children, we systematically evaluated the impact of maternal diabetes on offspring weight. No significant main effect of diabetes was found in either age group (Model I: Estimate -0.0241, *p* > 0.05; Model II: Estimate -0.0303, *p* > 0.05). However, a weak yet statistically significant positive interaction was observed between diabetes and month-of-conception squared in Model I (*p* < 0.05). Notably, both models showed a highly significant positive association with neonatal body weight (*p* < 0.001).

Beyond maternal diabetes, birth month and its squared terms consistently exhibited highly significant linear and non-linear effects on offspring weight, characterized by a declining weight trajectory as birth month advanced. Furthermore, female newborns consistently demonstrated a strong negative correlation with weight in both models. Additionally, higher parity (negative correlation) and advanced maternal age (positive correlation) exerted consistent effects across both models.

The models demonstrated excellent explanatory power, with marginal and conditional R^2^ values exceeding 0.80 and 0.95, respectively, indicating that the incorporated fixed and random effects effectively captured the overall variation in offspring weight.

In conclusion, while the primary effect of maternal diabetes on offspring weight did not reach statistical significance within the 3–36 and 3–78 month intervals, a subtle interaction effect trend was detected in the broader 3–78 month range.

## 4 Discussion

### 4.1 Adverse effects of GDM on pregnancy outcomes

GDM exerts a notable influence on delivery complications and neonatal outcomes. This study reveals statistically significant associations between GDM and various obstetric factors, including delivery mode, perineal status, preterm birth, postpartum hemorrhage, placenta previa, extremely low-birth-weight infants, macrosomia, neonatal infections, asphyxia, pathological jaundice, and hypoglycemia. Moreover, GDM exhibits strong correlations with the offspring's birth length, birth weight, and 1-minute Apgar score.

In the present study, we observed an unexpected inverse association between GDM and certain adverse outcomes, including preterm birth (*OR* 0.649), postpartum hemorrhage (*OR* 0.718), and placental abruption (*OR* 0.450). At first glance, these findings seem contradictory to clinical expectations. However, this phenomenon is likely attributable to indication bias and competing risks frequently encountered in large obstetric cohort studies (10). Women diagnosed with GDM are typically managed with intensive antenatal surveillance and receive earlier obstetric interventions. Consequently, spontaneous preterm labor is often preemptively terminated by elective induction or planned cesarean delivery before reaching 37 weeks of gestation. Similarly, close monitoring may allow for the timely management of potential hemorrhage risks. Therefore, rather than reflecting a true biological protective effect of GDM, these negative associations likely reflect the efficacy of enhanced clinical management in this high-risk population.

Previous studies have shown that maternal hyperglycemia is linked to adverse outcomes such as infections, macrosomia, and neonatal adiposity (11). Muche et al. (12) emphasized that GDM patients face higher risks of adverse maternal and fetal outcomes, including cesarean sections, premature rupture of membranes (PROM), and antepartum/postpartum hemorrhage.

Recent evidence indicates that a hyperglycemic intrauterine environment can result in a variety of unfavorable consequences for fetal development, such as an elevated likelihood of delivery complications and neonatal morbidity. Consequently, it is essential to prioritize the management of high-risk pregnant women, especially those with GDM, to mitigate these associated risks.

### 4.2 Effects of diabetes during pregnancy on children’s physique

The distributions of body length/height and weight among the two groups of children exhibited a trend of consistency, indicating a specific convergence pattern.

In summary, offspring growth is shaped by a complex interplay of genetic (e.g. parental genes) and environmental factors, including ethnicity, social environment, feeding practices, and family lifestyle behaviors (e.g. diet, exercise, and sleep) (13, 14). The influence of postnatal care is particularly evident in infants with low-birth-weight indicators (e.g. preterm or malnourished newborns), who often receive greater parental care and nutritional support. Although this promotes catch-up growth and helps them reach developmental milestones comparable to peers (15), it concomitantly elevates the long-term risk of overweight and obesity. Supporting this, Martin-Calvo et al. (16) observed that low-birth-weight infants tend to manifest compensatory growth patterns during later development.

Conversely, families of macrosomic infants proactively modify feeding practices to mitigate the risk of excessive weight gain, thus reducing overall developmental disparities. Nevertheless, this retrospective cohort study has certain limitations, especially in its inability to gather comprehensive information regarding home-based feeding practices and family environmental factors. Although we have now reported major outcomes separately for GDM and PGDM, certain secondary analyses relied on the combined diabetes group due to sample size constraints in the PGDM subgroup. This limitation should be addressed in future multicenter studies with greater numbers of PGDM pregnancies. Furthermore, only anthropometric outcomes were assessed, without metabolic or neurodevelopmental follow-up.

Several limitations inherent to the retrospective study design warrant acknowledgment. Most notably, we were unable to stratify the GDM cohort by glycemic control severity. In clinical practice, patients with lifestyle-controlled GDM (A1GDM) and those requiring pharmacotherapy (A2GDM) represent distinct risk profiles, with the latter typically exhibiting a higher burden of adverse perinatal outcomes. Future prospective studies with protocolized glucose monitoring and clearly defined severity categories are necessary to delineate these threshold effects.

This study has several critical limitations inherent to its retrospective, single-center design. First, we lacked standardized adiposity indices such as BMI-for-age z-scores, body composition, or growth SDS. Our reliance on absolute anthropometrics precludes differentiation between healthy linear growth and increases in adiposity, the latter being the more biologically relevant outcome for GDM-related metabolic programming. Second, we could not control for genetic growth potential, as mid-parental height data were unavailable; this omission likely confounds our assessment of “growth normalization.” Third, postnatal environmental variables—including breastfeeding duration, physical activity, socioeconomic status, and dietary patterns—were not systematically recorded, leaving us unable to separate intrauterine effects from potent postnatal modifiers.

Owing to these constraints, our findings are descriptive rather than mechanistic. The observed anthropometric convergence should not be overinterpreted as evidence of limited long-term metabolic impact. Subclinical insulin resistance, beta-cell dysfunction, or visceral adiposity may persist and progress despite normalization of linear growth. Future prospective studies incorporating direct assessments of adiposity, parental phenotyping, and detailed postnatal environmental audits are required to draw robust conclusions regarding the metabolic legacy of GDM exposure.

### 4.3 The significance of early screening and comprehensive management

While pre-existing diabetes in pregnancy is predominantly type 2, GDM represents the majority of diabetes-related pregnancies. First-trimester glucose screening is strongly recommended to identify GDM risk, particularly in high-risk populations—including women with advanced maternal age, pre-pregnancy abnormal BMI, or comorbidities like polycystic ovary syndrome (PCOS). Early detection and timely management of GDM can significantly reduce its incidence, thereby improving both maternal and fetal outcomes (17).

Optimized metabolic management before and throughout gestation confers substantial benefits to both mother and child. Evidence indicates that standardized GDM care—integrating dietary counseling, physical activity, and judicious pharmacotherapy—reduces adverse perinatal outcomes by stabilizing gestational glycemic levels (18). Self-management and consistent prenatal follow-up remain cornerstones of antenatal care (19). Furthermore, theory-driven interventions, such as personalized health education and the knowledge-attitude-practice (KAP) model, have been shown to improve maternal self-efficacy, yielding positive long-term effects on the health of mothers and their offspring.

GDM encompasses multiple domains, such as metabolism, endocrinology, nutrition, and psychology. A single discipline is unable to comprehensively meet its management requirements. The role of multidisciplinary team management in GDM is of critical importance. Studies have shown that collaborative care can lead to improved patient outcomes and a reduction in complications (20). Considering the substantial variations in maternal risk factors, disease severity, and lifestyle differences among patients, collaborative efforts across disciplines are essential for formulating personalized intervention plans. Through comprehensive and standardized medical interventions, the management of GDM pregnancies can be enhanced.

## 5 Conclusion

Diabetes during pregnancy is associated with a significantly higher incidence rate, substantially impacting pregnancy outcomes and markedly increasing adverse risks for maternal, fetal, and neonatal health. During fetal development, GDM significantly influences the birth outcomes of offspring, encompassing birth weight and length, accompanied by potential risks of macrosomia and post - delivery hypoglycemia. Nevertheless, these disparities gradually diminish during the subsequent growth process in childhood, indicating a convergence tendency in early growth patterns.

In conclusion, diabetes mellitus during pregnancy significantly exerts an impact on both maternal and fetal outcomes. High-risk pregnant women encounter elevated risks of complications, along with potential long-term health concerns for both the mother and the child. It is of utmost necessity to strengthen the screening and management of such cases across the entire pregnancy continuum.

## Data Availability

The original contributions presented in the study are included in the article/supplementary material. Further inquiries can be directed to the corresponding authors.
